# Men, maternity and moral residue: negotiating the moral demands of the transition to first time fatherhood

**DOI:** 10.1111/1467-9566.12138

**Published:** 2014-08-05

**Authors:** Jonathan Ives

**Affiliations:** Medicine, Ethics, Society and History, University of BirminghamBirmingham, UK

**Keywords:** fatherhood, antenatal, moral residue, maternity, involvement

## Abstract

This article discusses men's transition to first time fatherhood, with a focus on the way they recognise various in-tension moral demands and negotiate an appropriate role for themselves. The findings are taken from a longitudinal study, drawing on elements of grounded theory, comprising a series of face-to-face and telephone interviews with 11 men over a 9-month period from the 12^th^ week of pregnancy to 8 weeks after the birth. The analysis focuses on men's feelings and experience of exclusion and participation, and their response and reaction to that experience. The findings present two descriptive themes, ‘on the inside looking in’ and ‘present but not participating’, followed by third theme ‘deference and support: a moral response’ that exposes the dilemmatic nature of men's experience and explains the participants’ apparent acceptance of being less involved. The discussion explores the concept of moral residue, arguing that while deference and support may be an appropriate role for fathers in the perinatal period it may also be a compromise that leads to feelings of uncertainty and frustration, which is a consequence of being in a genuinely dilemmatic situation.

## Background

Recent literature has emphasised the complex normativity of early fatherhood. Miller (2011a), for example, characterises early father involvement as a socially constructed performance ‘set in a complex of changing expectations and norms of behaviour’ (p. 176) and Dermott (2008) proposes ‘intimacy’ as a way of conceptualising contemporary fatherhood as a:

Relationship that allows for an emphasis on the aspects of male parenting that fathers themselves view as most significant; emotions, the expression of affection, and the exclusivity of the reciprocal father-child dyad. (Dermott 2008: 143)

For Dermott, intimacy can bridge the ‘asynchronicity’ (Rotundo 1985) between fathering culture and conduct, providing scope for moving ‘beyond narrow formulations of fathers, either as failing to contribute to, or as sidelined from, family life’ (Demott 2008: 143) because they cannot, often for economic reasons, participate in the day-to-day practice of childcare. Economic drivers are significant here and, for Dermott, the pressing need for many fathers to undertake paid work mitigates and justifies, to some extent, a fatherhood that is premised on ‘caring about’ rather than ‘taking care of’ (Lewis 1995). Chin and Daiches *et al*. (2011) have found that feelings of exclusion and non-participation can be amplified on a father's return to paid work as the father's exposure to the day-to-day work of childcare diminishes. Genesoni and Tallandini (2009) warn that when men struggle to reconcile their personal and work-related needs with those of their families, they:

Frequently withdraw from this conflicting situation into their traditional role and do not attempt to change their habits; therefore they end up providing little support to their new families in terms of practical help. (Genesoni and Tallandini 2009: 316)

Responding to this complex picture, strategies to help fathers become involved and engaged with their children abound. While such strategies are ostensibly premised on improving outcomes for mothers and children, Collier (2010) reminds us that these:

initiatives have been shaped... by more than a rethinking of the effects on children of father involvement. Underscoring these developments have been beliefs about gender and personal responsibility, justice, equality and social cohesion. (Collier 2010: 142)

The policy rhetoric is, however, clearly focused on mother and child outcomes and is framed in terms of helping men develop ‘good’ fathering practices that preserve forms of acceptable fathering, to the benefit of the child, the family and society. This is clearly visible in antenatal and maternity health care, which is described in the Midwifery 2020 report as a golden opportunity for midwives to offer information, advice and support to fathers (Department of Health, Social Services and Public Safety 2010: 17). Others note the ideal placement of midwives to engage with and support expectant fathers (Deave and Johnson 2008). Nonetheless, a recent meta-synthesis of fathers’ experiences of pregnancy, birth and maternity care found that practitioners may not be well equipped to do this, arguing that more needs to be done to facilitate ‘authentic’ involvement of new fathers to ‘effect an active transformation to positive fatherhood’ (Steen *et al*. 2012: 9). This diagnosis is accepted by the Royal College of Midwives (RCM), whose recent guidance (2011) states that more must be done:

Since fathers are important influences on mother's health choices and experiences before, during and after the birth, it benefits the whole family when maternity professionals make fathers feel welcomed and involved and prepare them for their role at the birth and afterwards. Expectant fathers need to be included in all aspects of maternity care and be offered opportunities to discuss their feelings and any fears they may have. (Royal College of Midwives 2011: 12)

Depending on what is sought from father involvement and/or engagement, their ‘successful’, ‘authentic’ and ‘positive’ involvement in the perinatal period cannot be defined solely in terms of the extent to which it brings about the proffered goods for mothers and children, but also the extent to which their involvement conforms to a particular normative agenda. The RCM statement appears to endorse a normative agenda loosely defined in the narrative of the new father which, notwithstanding salient criticism that it is neither clearly new nor routinely practised (Burgess 1997, Craig 2006, LaRossa 1988, Lewis and O'Brien 1987, Rotundo 1985), is characterised by the key concepts of physical presence, emotional connectedness and an equal division of responsibility for child-rearing and domestic labour (Collier 2010, Collier and Sheldon 2008, Deinhart 1998, Dermott 2008, Henwood and Procter 2003, Ives *et al*. 2008, Lloyd 2003, Sullivan 2000). It also draws on the phenomenon of intensive parenting, which has been much discussed and critiqued in the literature (Furedi 2002, Lee and Bristow 2009, Lee *et al*. 2010). While Shirani *et al*. (2011) argue that, by appealing to a gendered valuing of autonomy and competition, men are able to remain relatively insulated from the demands of intensive parenting, key features of that discourse are clearly evident in the job description of the new father, and certainly in the context of defining appropriate behaviour for expectant fathers. These behaviours include, among other things, taking time off work to attend antenatal appointments and scans, engaging in household refurbishment, making financial provision for the family, giving emotional and physical support, shopping for the baby, participating in screening decisions and antenatal education, co-creating birth plans, becoming educated about labour and the signs of labour, and being included in all aspects of maternity care.

While the transition to fatherhood is increasingly understood as a relatively unstructured and ambiguous process (Chin *et al*. 2011, Doucet 2009, Draper 2002, 2003, Genesoni and Tallandini 2009, Miller 2011b), relatively little attention has been paid to the way in which the navigation of this complex journey affects, and is affected by, men's engagement with health services. Discrete studies looking at screening, labour and birth are numerous (Chandler and Field 1997, Dheensa *et al*. 2013, Draper 1997, 2002, Longworth and Kingdon 2011, Premberg *et al*. 2011, Vehvilainan-Julkunun and Liukkonen 1997) and the fact that men often feel sidelined is well established (Chandler and Field 1997, Deave and Johnson 2008, Draper 1997, Jordan 1990, Vehvilainan-Julkunun and Liukkonen 1997). Given that health services are used to help men make a ‘positive’ transition to fatherhood, and given the implicit normative agenda that dictates what a positive transition is, it is important to understand to what extent men's own perceptions of their role as fathers mirror or challenge the normative agenda adopted by the RCM and others, and in what ways that agenda might be challenged or reinforced by men's interaction with health services. The data presented in this article were collected during an Economic and Social Research Council study carried out as part of a broader reflexive bioethics project exploring the ethical normativity of contemporary fatherhood and the role of health services in responding to and shaping those norms (Draper and Ives 2009, 2013, Ives 2013, Ives *et al*. 2008).

## Methods

Interview data were gathered between September 2010 and September 2011 from men becoming first time fathers. The men were followed over approximately 8 or 9 months (from the first 12 week scan to 8 weeks after the birth). Regular face-to-face and telephone interviews were conducted over this time, which explored the participants’ experiences and feelings about becoming a father. Ethical approval was obtained from West Midlands-Solihull National Research Ethics Service committee prior to commencement (ref: 10/H1206/41).

Inclusion criteria were men who (i) were becoming fathers for the first time and (ii) could speak conversational English. The only exclusion criteria were an inability or unwillingness to consent. The sampling of those who met the inclusion criteria sought to achieve maximum demographic variation, with a balanced mix of ethnicity, education, age and employment. A recruitment target of 10 men was considered appropriate and feasible, given limitations of time and funding, and the large amount of data expected from each participant.

The men were recruited through community midwives and maternity units in the West Midlands, UK. The midwives provided potential participants with an information pack (directly or via their partner) at the booking appointment, which directed interested men to contact the author. The men were offered £150 in supermarket vouchers as a reimbursement for their time, an amount agreed with the NRES committee to be appropriate. In all, 22 men expressed an interest. Attempts were made to contact all 22 respondents and contact was established with 20. Four did not meet the inclusion criteria. Of the remaining 16, one chose not to participate. Eleven men were purposively sampled from the remaining 15 to achieve as wide a demographic variation as possible (Table1). The four men not sampled were all tertiary educated, professional white men and their inclusion would have made the sample too homogenous.

**Table 1:** Participant demographics (self-reported)

Pseudonym	Ethnicity	Age	Relationship to child's mother	Highest educational qualification	Employment status	Current/most recent employment
**Peter**	White British	58	Married	O'level	Retired	Haulage
**Janesh**	British Aisian	27	Married	Vocational degree	Employed	Dentist
**Ben**	White British	22	Co-habiting	A-level	Employed	Customer service
**Daniel**	White British	31	Married	Degree	Employed	Civil servant
**James**	White British	29	Married	Degree	Employed	Project manager
**Charles**	White British	40	Married	Degree	Self-employed	E-commerce
**Michael**	White British	22	Cohabiting	NVQ	Employed	Marketing
**Sandeep**	Indian	31	Married	Vocational degree	Employed	Optometrist
**Brian**	White British	44	Married	NVQ	Self-employed	Mortgage advisor
**Simon**	Black Caribbean	35	Married	Degree	Employed	PR account manager
**Phil**	White British	35	Married	HND	Employed	IT

**Figure 1:**
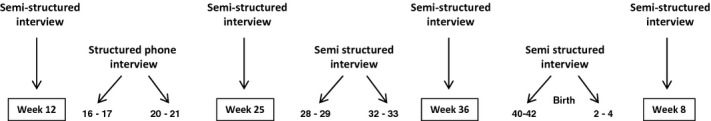
Placement of interviews.

A combination of in-depth face-to-face and telephone interviews was employed, with one interview conducted approximately every 4 weeks (Figure1). Although longitudinal data were collected, the nature of these data is not explored in this article. The telephone interviews were relatively structured, aiming to elicit information about the ongoing routine or unusual events that had occurred since the last interview. The face-to-face interviews were semi-structured. The topic guide comprised a series of standard questions broadly based on those asked by Miller (2005) in her study of the transition to motherhood. Standard questions focused on the participants’ expectations, intentions, perceptions of self and others, birth, normative ideals of fatherhood, experience of maternity services, difficulties or concerns, support and information needs. The topic guide was also informed by the content of previous individual telephone interviews, enabling face-to-face interviews to be oriented around issues raised by participants themselves.

The interviews were arranged at a time convenient to the participants, ranging from early morning to late evening. The majority of the face-to-face interviews were conducted in participants’ home, with a small minority conducted in the author's office or home at the participants’ request.

All the interviews were transcribed verbatim by a contracted transcription service. Data were analysed by the author, and ongoing and concurrent data collection and coding facilitated a reflexive analysis grounded in the data. An explicitly reflexive analysis helped to ensure that the pre-existing relationship between myself as analyst and the topic was interrogated. I had a longstanding interest in the ethics of fatherhood and families, but my specific interest in the relationship between the transition to fatherhood and health services was motivated initially by my own experience of becoming a father. Additionally, over the study period I was expecting my second child. It was important to ensure that my own current and past experience did not take precedence over that of my participants. Finlay (2002) emphasises the importance of (i) examining the impact, position and perspective of the researcher; (ii) promoting insight through examining personal responses; and (iii) opening up unconscious motivations and implicit bias in the researcher's approach. Maintaining a reflexive diary helped me identify and expose any bias in my interpretation of the data, which could then be consciously challenged and if necessary revised. Being aware of my own experiences and expectations helped me to avoid asking leading questions during interviews but it was also possible to draw on my own experiences to build rapport, show solidarity and establish trust through reciprocal sharing. Reflexivity was particularly important in the context of this research, given the temptation to focus only on those data that supported my already published opinions (Draper and Ives 2009, 2013). To help manage this risk, and in addition to conscientious reflexive practice (Ives and Dunn 2010), emergent themes from initial interviews were explored in subsequent interviews, enabling a form of iterative ‘member validation’ in which participants were invited to reflect upon the ongoing analysis of the dataset as a whole and my interpretation of their own individual story. Additionally, my initial and ongoing findings were shared with the steering group, where they were examined and critically discussed by fatherhood scholars, qualitative researchers and practitioners. Disagreements occurred and alternative interpretations were explored.

While the study did not explicitly follow a specified grounded theory methodology, the analytic process can be aligned with grounded theory (Corbin and Strauss 2008) more than any other. Initial and open coding (Saldana 2010) broke the data down into discrete parts (using NVivo), allowing a close examination of content. A coding list and rationale was maintained to ensure consistency. As codes were formed they were grouped into descriptive subcategories through a process of axial coding, which sought to reassemble the 1034 initial codes into manageable units. Theoretical and selective coding began early on and ran alongside initial and axial coding, with the generation of analytic memos that captured emerging ideas about relationships between codes, categories and subcategories. Using those analytic memos as a starting point, categories and subcategories were interrogated formally, with the use of mind maps to explore and theorise the relationships between them (for example, between accounts of gender obligations and concepts of justice and rights; between the perceived purpose of maternity health services and men's role in the process). This process began to generate an overarching set of core themes (or theoretical codes (Charmaz 2006)) and develop explanatory hypotheses. Constant comparison and deviant case analysis (Silverman 2005) were employed throughout to rigorously test emerging hypothesis, which was particularly important if the participants appeared to confirm something I was expecting or wanting to see.

The interpretative analysis was informed by three theoretical assumptions. Firstly, following Miller (2011a), it was accepted that fathering can be conceived as a performance that is both complex and normative. Secondly, it assumed a Bourdieusian straddling of structure (social physics) and agency (social phenomenology), which function as ‘double-focus analytic lenses that capitalise on the epistemic virtues of each... while skirting the vices of both’ (Bourdieu and Wacquant 1992: 7). Thirdly, it assumes a moderate pragmatic ethical naturalism (Racine 2008) that assumes the study of morality in practice can and should inform normative ethical theorising in a meaningful way (Frith 2010, Ives 2013).

## Findings

This section presents three core themes identified in the data. The first two, ‘on the inside looking in’ and ‘present but not participating’ are predominantly descriptive, outlining the participants’ experiences of feeling separated, sidelined or excluded, to varying degrees, and their reactions to that experience. There is tension evident in these themes, which was unexpected. Fathers feeling uninvolved and excluded is commonly reported in the literature, but is normally portrayed as something negative that leaves fathers unsatisfied and lets families and service users down. The fact that these data suggested that some fathers may be content with less involvement than norms dictate led me to ask whether there was anything specific in the experience of these fathers that might explain that acceptance.

The question this led me to ask was whether or not these men were acting wrongly in accepting a kind of involvement that falls below that dictated by the current norms of contemporary fatherhood. The data were therefore further interrogated for concepts, thoughts and ideas that could make sense of and explain these phenomena. The first two themes were taken as central concepts, and mind maps were developed that began to explore potential relationships between those concepts and other existing codes and categories. This led to the development of the third and final theme presented in this article, ‘deference and support: a moral response’, which tries to make sense of the participants’ reactions and responses in a way that is consistent and coherent with the whole dataset.

### On the inside, looking in

One defining feature of participants’ experience was feeling they had a central and important role to play but simultaneously feeling separated and apart. This apartness tended to be experienced as a feeling of relative distance between themselves and their child, generally coupled with a feeling of their relative unimportance. Simon, for example, talked about feeling that his wife is ‘that tiny bit more ahead’ of him. The presence of a direct physical relationship with the child was, for Simon, essential to parenthood properly conceived, and this was something he lacked:

I think she's that tiny bit more ahead because it's natural, she's physically feeling the changes, she's the one taking the strain regards carrying this child so she's building... I can see her building that affinity with it and for me obviously not... I think about myself as preparing to become a dad but you can see with her, she's becoming a mother, that's the difference. (Simon, face-to-face interview 1, week 14)

Simon's description of himself ‘preparing to be a dad’, when set against the assertion that his wife is ‘becoming a mother’, portrays his wife as an active protagonist, while narrating for himself a less active role, waiting to develop a physical connection to the child that will turn him into a father. Similar language was used by Phil, who felt removed from the pregnancy because he did not experience daily physical interaction with the developing child. While cherishing the moments when he did physically connect, those moments also acted as reminder of the distance that usually exists between them:

She's rubbing him every day and she's got that contact and she feels him kicking all the time. So yeah, I'm removed from that, aren't I? And I think, as I said to feel that, him kicking his dad, kind of yeah, definitely gave me that physical contact that [wife] is probably quite used to. But it is quite distanced because you're not... you're not developing the baby are you? (Phil, face-to-face interview 2, week 14)

### Present, but not participating

All the participants reported being satisfied with healthcare services and their interaction with healthcare professionals. Their overall satisfaction tended to be outcome based, and provided their partner and child were looked after they had no complaint. This overall satisfaction, however, does not equate to a feeling of involvement or engagement with or by health services, but merely that their involvement matched their expectations and was congruent with, and reinforced their feeling that at this stage pregnancy and parenthood were things that can be observed and supported but not participated in. Ben, for example, sometimes felt in the way, and described trying to talk to the midwife in his partner's absence. He reported being ignored, and felt that ‘obviously I'm not supposed to speak in those situations’. He continued:

[W]e're expected to do a lot more these days, we're expected to be a lot more involved... but when we are involved we're still a bit on the outskirts from what I've seen... I don't know if it's because a lot of women don't take their partners with them, I'm not sure how other people work. It's sort of maybe they're not always used to having a man there as well, but it would've been nice to be acknowledged a little bit more, just so you feel a bit more part of it more than anything. Because you feel a bit awkward sometimes just stood there like ‘Should I wait outside?’ (Ben, face-to-face interview 1, week 15)

Others described events that implied they were surplus to requirements or interlopers in a private space. Phil described wanting to record the baby's heartbeat on his phone but being initially prevented from doing so because the midwife pulled a curtain across. This physical barrier between himself and his wife made him feel like he ‘shouldn't be looking’:

I felt as if I shouldn't be looking kinda thing, you know, ‘coz when you grow up and people pull the curtain across, it means you shouldn't be looking in there doesn't it or it's like a private area. Even though it's my wife, I'm kinda thinking, I think I might have even backed into the err, behind the curtain when I went to record off the – off the machine on the wall. But it made me feel quite uneasy to be honest. (Phil, telephone interview 2/3 combined, week 21)

For Phil this physical barrier replicated and reinforced the natural barrier to participating in pregnancy that he experienced as a man. While he was able to overcome this by asking permission to make the recording (which he was given), the initial drawing of the curtain took on a special significance for Phil because he had brought with him into the healthcare encounter a certain set of expectations and experiences that encouraged him to interpret it as a barrier intended to mark the area out as private, and keep him at distance.

While no men welcomed feeling excluded, none reported it as a problem that impacted on their experience and many saw it as entirely appropriate and consistent with their fatherhood performance. Brian, for example, felt he did not need to be actively included by health services, as midwives were there purely to look after the mother and baby:

I'd say the service is there for the mother. At the end of the day they're the ones having the baby. And rightfully so. I wouldn't say it's wrong [that fathers are not actively involved by the midwife]. It's nice for us to be involved in the whole process. But at the end of the day, let's face it, once a woman is pregnant, they don't need us, well, the NHS, not the wife. (Brian, face-to-face interview 3, week 38)

Sandeep felt that being excluded, and sent away from the hospital after the birth of his daughter was an appropriate preparation for fatherhood. He reasoned that he would have to leave his family to earn a living, so he may as well start getting used to it:

I think as soon as... we had her basically, it was kind of, you know, at the hospital they said ‘Oh, you have to leave at 8 o'clock’, so it's kind of like you always seem to be sent away as the father anyway, so I don't know, maybe it's just a – I don't know, sort of getting into [my] head now, I'm going to have to leave her for periods. It's like, you know, it's I've got to start earning a living and stuff like that. (Sandeep, final face-to-face interview, 7 weeks post-birth)

Charles appeared very relaxed about not actively participating during appointments. When it was put to him that some other men felt a little excluded, he gave the following explanation for his relative insouciance:

Yeah, but at the same time you kind of have to think, okay yes we're in it together, but actually, this isn't about me, this is about this lady, you know, she's the one that's going to go through it, she's the one that's going to have to you know, push it out, and she's the one whose body is going to change beyond belief... I know hormones will be up and down, I know she'll be upset for something which could be nothing to me but massive to her... Sidelined? I suppose actually with the midwife in that first session, it was kind of, everything was you know, aimed at [my wife] and I could have just been a fly on the wall, erm, but I wasn't there really because of the midwife, I was there because of [my wife], I need to hold her hand. (Charles, face-to-face interview 1, week 12)

### Deference and support: a moral response

Perceiving a distance between themselves and the pregnancy (and subsequently the child) as something integral and unavoidable to the situation, the primary roles described by the participants during the pregnancy drew heavily on masculine ideals of the stoical protector. This was expressed in various ways, ranging from very physical protection such as making sure their partner does not do physical labour, eats properly and is not bumped about by crowds, to more emotional protection. Talking about the latter, Ben described ‘putting on a brave face’ and making sure he is there to support his partner:

[S]he's in a bit of a sorry state, it's not nice to watch. You can't help but feel a little bit responsible because she wouldn't be in the situation without me, but I think I've got to stay positive and try to make sure that, not so much put on a brave face, but just make sure I'm there to support her more than anything. It's no good both of us having a bit of a nervous breakdown. (Ben, telephone interview 4, week 32)

The significance of the protector role appeared to be amplified by men's perceptions of their partners becoming more vulnerable as result of pregnancy but the ambition to protect was simultaneously frustrated by the feeling that they had limited capacity to fulfil this role. Simon spoke of his wife becoming less resilient, and a growing feeling of ‘uselessness’:

As I say, for me it's [wife]'s health, and particularly not being able to do bugger all about [it] and I think that's something... I can imagine that's something that most men feel and I'm sure are feeling the closer you get and once we actually get to [wife] actually giving birth as I say I feel... when [she used to be] strong and resilient to everything, knowing that she's now not resilient to anything... She's really knackered, she's now starting to feel things that she didn't feel before, you know she's starting changes in her body that she's not happy about, she's getting bigger and she can't do anything about it and I'm watching her saying all this and thinking I can't do anything to help you, I'd actually say this is... well it's not awful but it's certainly, for her, I mean for her it's got tougher therefore for me, I'm just feeling that little bit more useless. (Simon, face-to-face interview 1, week 14)

Janesh echoes this frustration at not being able to help his wife during a severe bout of pregnancy-related illness in the first trimester:

I didn't know how to help her. And I think that's frustrating. Really frustrating where you can't, you can't do anything. And, you know, you try and do everything you possibly can, you know, make sure she's eating the right things, used to sit there reading the Internet trying to, you know, what can make her better, and speak to as many people, mums and stuff, and see, you know, what can – but she was going – I think it was really hard for her, she had the worst part of it. (Janesh, face-to-face interview 1, week 12)

Both Simon and Janesh were at pains to emphasise that their partners were having a worse time than they were, and sought to pre-empt and deflect any idea that they thought their own experience was comparable or equally important. Generally, the men in this study appeared to be attuned to the moral risk of expressing disquiet or discomfort about their own situation. They felt that their own problems could not be compared to the experience of their partners and that complaining would be inappropriately self-centred.

The extent to which these feelings of frustration and helplessness are tied to the context of pregnancy and fatherhood, or whether they are a feature of the general experience of watching a loved one suffer, is not clear. It is likely there is a difference, at least phenomenologically, for two reasons. Firstly, in pregnancy, a man has some responsibility for his partner's condition (a feeling articulated by Ben above), which may suggest he has an obligation to try to offer some relief. Whether this is reflective of a concern for the ‘other’ or a need to assuage the feelings of guilt that are connected to this responsibility is a question about which these data can, unfortunately, say nothing. Secondly, the men in this study were willing participants in a normative discourse of new fatherhood that emphasised joint experience, joint responsibility, togetherness and sharing in the trials and tribulations of pregnancy. The expectation of a shared experience may make it harder to watch one's partner suffer alone.

The protector role extended, for some, to the unborn child. This point was made explicitly by Michael who saw this as form of proto-fathering. For Michael, being a protective partner was part of being a father. By protecting the mother, and looking after her interests, you are also doing what is in the best interest of the baby, which he described as ‘kind of being a father’:

[Y]ou know, at the end of the day, the mother is carrying your baby, so you, kind of, it's in the best interests that the mother looks after herself, so by helping her as much as you can and protecting her, you're, kind of, being a father to the baby in a weird kind of way, if that makes any sense? (Michael, face-to-face interview 1, week 12)

Many participants recognised that fully enacting the protector role required a level of control over their partner they felt was inappropriate. Daniel, for example, spoke about wanting to keep his partner safe, and expressed disquiet at her choice of transport to work in the third trimester. Nonetheless, he tended to ‘defer to her’, and saw his role as to ‘sort of support her in decisions which she makes rather than try to launch her too much in one direction or another’. He justified this by appealing to her more intimate knowledge of, and contact with, the pregnancy:

[S]he knows how she's feeling. Erm, you know, she knows how tired she's feeling... whether she wants to take the bus or cycle. Er, you know, she knows what she... wants to be eating... you know, yeah, she's in... fuller contact with the situation than I am. So I would defer to her on those grounds. And yes the, you know, medical side is a factor as well. But, you know, I think that even if she wasn't medical then, you know, I would still say, you know, ‘hands off I'll, I'll let you decide what's best for you’. (Daniel, face-to-face interview 2, week 25)

The obligation to protect, however enacted, was thus tempered by a concurrent feeling that fathers ought not to exert control over any aspect of their partner's pregnancy. There was a sense in which the participants considered their partners the rightful decision-makers, justified by a combination of their more intimate embodied knowledge of the pregnancy and the child, appeals to bodily integrity, a complex (but unarticulated) construction of desert, justice and rights and a pragmatic desire to avoid conflict. Peter combines many of these justifications when describing his acceptance of his wife's decision not to breastfeed, despite strongly disagreeing with that decision:

I'm not pressurising [wife] at all. I think she realises that I don't really agree with her, erm she might do it and she might change her mind at the last moment. And I'd be all for that. But I can understand, you know, her fears. So that's not going to be an issue... [She]'s the one having the baby, I'm the father, I've really got the easy part. So why should I put any pressure on her to do something that she doesn't want to do, erm cause a strain between us, because if I go adamant, you know, I really want you to do this and this, and she gave in because I was pressurising her, there would be resentment. And maybe she'd agree that I was right in the end, I don't know. It's just not worth putting any strain on the relationship. She's going to be very emotional in any case, she's going to be a new mother so, no, there's no point, it's her choice, I wouldn't want it any other way. (Peter, face-to-face interview 3, week 36)

Another reason for adopting a supportive and deferential role was given by Ben. Talking about his partner's reluctance to have a C-section, he felt her reasons for resisting it were poor, but told her ‘it's completely up to you what you wanna do ‘coz it's your body at the end of the day. I can't tell you what to do with it’. He worried, however, that he was failing to do the right thing by not taking a critical stance, wondering aloud whether his partner would ‘rather I was a bit more, like, had a bit more of a firmer input’. It was also clear he did not want to put pressure on his partner because he did not want to be held accountable if anything went wrong. By staying out of active decision-making he could avoid responsibility if things went wrong:

[I]f anything was to go wrong, then I'd feel responsible as well, which I suppose is quite a selfish way to look at it but... I wouldn't want that on my own mind really, let alone I wouldn't want her to think it (Ben, face-to-face interview 3, week 37)

While Ben appears to chastise himself for being selfish and may be acting wrongly by avoiding responsibility, he experiences a genuine moral dilemma. He feels he ought to offer his opinion and help guide his partner to the decision he feels is correct, but he also feels obliged to acknowledge her absolute right to control her own body. His concern that he would be held responsible if anything were to go wrong played heavily on his mind and tipped the balance in favour of remaining neutral. This decision may be interpreted as moral weakness but his unwillingness to be responsible in this context is not necessarily indicative of moral failing. This is because his difficulty is compounded by his feeling that it is not appropriate for him to have that responsibility. If he did offer an opinion in favour of a C-section, and something went wrong, he would not only be responsible for that outcome, but that responsibility would be a direct consequence of his having usurped power and made a decision that is not his to make. What Ben did not appear to consider is a potential middle way, in which options could be discussed and negotiated without him taking control. He saw his partner's right to control her own body as absolute and was unwilling to attempt any negotiation for fear of transgressing that absolute boundary. His preferred solution was withdrawal but it clearly made him uncomfortable.

The overriding sense of the mother having a natural authority over pregnancy and child-rearing decisions was illustrated forcefully by James, who became a stay-at-home dad, and described how his routine for their daughter was disrupted when his wife returned home. At weekends and after work, he said, ‘the boobs take over’, meaning that when his wife is around she was able to override the routine he was trying to establish:

[W]hen [my wife] hears [our daughter] crying... she just sort of wants to pick her up, feed her and comfort her, whereas, you know, I'm trying to get her used to a bit sorting herself out, as it were... At the weekend... the boobs take over when [our daughter] starts crying, whereas, you know, I'm just like, ‘It's not that, it's not that’. But yeah... I think it is pretty much a bit more, well we are a bit more unattached with things because we haven't got that physical bond... of breast feeding and that I think... when [my wife] gets home... from work and even if she's just had a feed and err, she cries ‘Oh, can I feed her, can I feed her, can I pick her up, can I hold her, cuddle her’, which is a bit annoying but then she doesn't settle well in the evening anyway, she's not an evening settler. Like Gina [Ford] says [babies] should be down by 7. She'd be lucky to be settled by about half nine. So whether that is ‘coz of [my wife] or whether that is ‘coz of [our daughter], I don't know. (James, final face-to-face interview, 8 weeks post-birth)

James rationalised and justified this taking over through an appeal to the physical bond his partner has, and needs to maintain, with their daughter. He did not feel this was something he should stand in the way of, yet it is clearly a frustration. It is, however, a frustration he feels obliged to live with. Simon expressed similar feelings. He often wanted to disagree with his wife, to push against her, but felt that he ought not. He could not explain why, just that it seems right:

Simon: Ultimately I always do what she says because she's, you know... arguably has the right to make those bigger decisions because currently she spends a lot more time in the house, for example, she's the main sort of person looking after our child at the moment and... those big decisions should probably fall under her... remit, but I sort of do challenge. Be interesting to see how that develops with a child and how that added responsibility, whether or not that tension would be become greater: hopefully not. But I think you sort of have to be aware of that there is always that tension, that you know she makes – she – we'll both make decisions collectively, I know that she probably has the upper hand in those decisions. But I can't help push that all the same.

Author Yeah... that, kind of, tension is one that I'm picking up throughout and... it's coming out as a theme of this research, that there's a sense in which fathers have got their own interests but tend to feel like mothers’ views carry a bit more weight because of the role that they play, they have a tendency to...

Simon Yeah, I mean I'm sure... there's plenty of men in the world who think that shouldn't be the case. You know, but probably, is it nice that men think like that? I don't know. But that's how I think as well. It's odd. Is it odd? No, it shouldn't... I can't explain it really. It's just a fair point I just can't – it seems right, why it's right I don't know. But it just seems right.

### Limitations and strengths

Before moving on to a discussion of the data presented above, one initial limitation to bear in mind is that the participants were a relatively small group of self-selected volunteers who wanted to talk about their experiences. They embraced fatherhood and loved and respected their partners, and this places immediate limits on the transferability of this analysis, as it is likely that men for whom the prospect of fatherhood is not welcome or who do not share that same broad moral outlook would not experience the transition in the same way. Furthermore, despite making attempts to recruit participants from a range of ethnicities, education and employment, the sample in this study mostly comprised well-educated white men who were in stable relationships with their pregnant partner. Nonetheless, this study presents an in-depth exploration of the transition to fatherhood from the perspective of men who are motivated to do well and are committed to fatherhood, which may well describe most men who become fathers. The small sample size made it possible to gather a great deal of in-depth data and in the process develop a personal rapport with participants, establishing a trust and mutual understanding that made possible intimate disclosures. The longitudinal data gathering made it possible to follow up on thoughts expressed and problems raised in future interviews, providing an opportunity not available in many studies of fatherhood to discriminate between issues of ongoing significance and transient issues that may dominate a single interview but be overall less significant.

Another potential criticism of this article is that it has not acknowledged or undertaken an analysis of the way that similar tensions may operate on mothers and motherhood. Motherhood is equally challenging, in ways similar to those described above but also in different ways (Miller 2005). It is beyond the scope of this article, however, to engage in any comparative analysis, suffice to say that the lack of a discussion of motherhood reflects the focus of the article rather than a lack of consideration or a belief that motherhood is any less complex. A similar study with mothers may be a valuable addition to the literature. Another valuable continuation of this work would be a repeat of this study with the female partners of pregnant women. This would allow an exploration of whether the findings reported here are a reflection of a specifically male experience or the experience of being the partner of a pregnant woman.

## Discussion

These data are not inconsistent with the notion of fathering and preparation for fatherhood as a performance (Miller 2011a) or, perhaps more accurately, a set of performances. What they highlight, however, is the way in which the transition to fatherhood can be experienced as a complex and confusing period in which different performances, driven by different and often conflicting values and norms, are negotiated, combined and constructed. It is a period of uncertainty, in which men experience the pressure of norms that are associated with the role of expectant fatherhood, but in which their sensitivity to the privileged position of their partners, and sometimes an effective marginalisation by healthcare workers, can lead to uncertainty and confusion about what precisely their role is, and even the appropriateness of claiming a defined role for themselves that is independent of, and unmediated by, their pregnant partner.

The men in this study were sensitive to the burden pregnancy placed on their partners and tended to feel that bearing that burden gave their partners the right to be decision-makers. Far from being resented, this was embraced and perceived to be legitimate and appropriate. Even when the men disagreed with their partners’ choices they were reluctant to oppose or persuade them, because to do so would transgress a moral boundary, representing a usurpation of power and control that is not legitimately theirs.

At the same time, the acceptance of this discourse can place men in a difficult position, in which they must negotiate a path between maintaining an appropriately supportive role that defers to the reality of embodied pregnancy, respecting their partners’ absolute right to bodily integrity, refraining from trying to exert power and control, seeking to be active and involved new fathers, expressing their own views and, overall, participating in ‘all aspects of maternity care’ (RCM 2011: 12). I have argued elsewhere (Draper and Ives 2013) that while men have a number of legitimate moral interests in their partner's pregnancy, including the interest in being a father to the foetus, the extent to which men can legitimately act to serve those interests may sometimes be limited by other trumping concerns. The data presented here suggest some men see bodily integrity, and the close physical connection between mother and baby after birth, as a trumping concern, which gives their partner absolute rights. This discourse dominates their thinking, to the extent that they need to renegotiate their views on what appropriate father involvement means in this context.

This renegotiation led many participants to accept and occupy a position of deference and support, premised on their partner's absolute right to bodily integrity and the belief that their partners have a greater claim to determine the narrative of the pregnancy. Exerting, or trying to exert, power and control over a pregnant partner was felt to be wrong, and the risk of committing this wrong is circumvented by stepping back and adopting a relatively subservient role. While few men in this study were willing to think of themselves as fathers proper until after the birth, this positioning represents the construction of a fathering role that was, for these participants, appropriate to the circumstances of pregnancy.

This was, for some, a compromise position that led to feelings of frustration and powerlessness. For others it was a compromise that represented an active and positive repositioning that enabled them to reconcile the multiple and competing moralities that made claims on them. In making this compromise the participants were able to construct themselves as good men because they were stoically accepting their lot and putting up with their own interests coming second without complaint or trying to make it ‘about them’. At the same time they could construct themselves as a good partner by demonstrating sensitivity to, and reproducing, feminist discourses that comprise the rejection of patriarchy and prioritising a discourse of bodily integrity. This compromise entails at least a partial rejection of the new father discourse, abandoning the notion of equal partnership and accepting a more secondary role that does not require involvement in all aspects.

The acceptance of a more subservient role of deference and support, while allowing men to act in a way that is consistent with what they perceive to be trumping moral concerns, creates something of a moral residue. Proponents of moral residue arguments, such as Williams (1965) and Marcus (1980), claim that when a dilemmatic situation is encountered the decision-maker, despite feeling they have acted correctly, experiences some feeling of guilt or remorse because in acting correctly overall they have failed to act correctly in some other respect. The lens of moral residue helps us make sense of the phenomena observed in this data. While participants who adopted this position considered it the right thing to do, the moral residue of this decision left them uncertain, questioning and in some cases frustrated and less than satisfied with their role.

The root of the difficulty, and the reason why some men experience a moral residue, lies in their acceptance of multiple and competing normative discourses, which they tend to frame in terms of absolute rights and obligations. The strong discourse of bodily integrity, evident in conversations with all the participants, was reproduced and reinforced during healthcare consultations that are centred on the mother and the mother's body. In tension with this is the discourse of new fatherhood, equally ubiquitous and expressed in terms of absolute obligations and summed up in the desideratum of the RCM and others that fathers should be involved in all aspects of maternity care. It is impossible to adhere absolutely to both these discourses, and so one has to find a compromise which, in some cases, may lead to some sense of moral distress because whatever one chooses one is failing to fulfil one's obligations elsewhere. Feelings of uncertainty, frustration and anxiety are all associated with moral residue and moral distress and, according to Epstein and Hamric (2009), can be associated with withdrawal from the distressing dilemmatic situation. With this in mind, we might begin to make sense of the phenomenon, observed by Genesoni and Tallandini (2009), that men who struggle to reconcile the competing demands of work and family often withdraw into more traditionally defined and less morally challenging roles, which arguably feeds into the persisting asynchronicity (Rotundo,^1985^) between fatherhood culture and conduct. Dermott (2008) found that economics is important here, and the analysis presented in this article suggests another piece of the puzzle may be moral residue created by viewing dominant moral discourses around fatherhood and pregnancy in absolutist terms, placing some men in a dilemma that cannot be neatly reconciled.

The extent to which these tensions are a ‘problem’ that requires resolution depends on the extent to which they lead to short-term or long-term difficulties for families, society or the individuals that comprise them, and this is something for future research to explore. It is, arguably, problematic if health services routinely reinforce and reproduce gendered discourses of parenting that exclude men and uncritically reinforce pre-existing beliefs that their role ought to be one of deference and support, which prevents them seeking the involvement they and their partners want. It is only wrong in itself, however, in so far as it appears to contradict the normative agenda of fathers having an authentic and positive involvement in all aspects of maternity care. Any wrongness depends entirely on how the concept of authentic and positive involvement is cashed out. For the men in this study authentic involvement was a negotiable concept. It was necessarily and, for them, legitimately, constrained by the medicalised nature of healthcare consultations (in which the focus is ‘properly’ on their partner's and child's health), and the belief that through the work of pregnancy their partner has earned the right to be the decision-maker (which appeals to discourses of bodily integrity and justice). It was partially constructed and partially discovered in practice, creating an authentic fatherhood that is tailored to each individual because it is created in the context of individual relationships and life courses. For the men in this study, the tension they experienced and the moral residue that sometimes resulted, was part and parcel of the process of becoming a father, which they dealt with in divergent, but arguably legitimate ways. Adopting a position of deference and support *is* authentic *–* and can be an appropriate and positive moral response.

An alternative interpretation is that what is described here is a path of least resistance in which men recognise that existing institutional and familial structures make certain kinds of compromise more or less achievable. It is possible that for some men the negotiation and construction of a fatherhood role is a less reflexive and active process than has been described above, and it may be more a product of external constraint than autonomous choice. Either way, and however we interpret the level of individual agency involved, the experience of the transition to fatherhood seems to involve, for the men in this study, both a reaction to external pressure (both normative and practical) and an internal striving to be a certain kind of father, partner and man.

## Conclusion

While it is limited in many respects, this study offers useful and important insights that may help us better understand how some men negotiate the transition to fatherhood and, importantly, encourages us to think critically about how that transition can be facilitated more generally. The golden opportunity (described in the Midwifery 2020 report) that exists here is not to actively involve fathers in all aspects of maternity care. Rather, if health services are going to be involved in helping men make the transition to fatherhood, they should be preparing, enabling and empowering them, where necessary, to engage in this moral negotiation, while recognising that many, if not most, will be capable of doing this independently. This moral negotiation may lead to involvement in only some aspects of maternity care, or partial involvement in all aspects, and lead to many different parenting arrangements in a compromise that is specific to the couple and their individual circumstances The danger of policies and recommendations that use the language of authentic involvement and involvement in all aspects of maternity care is that this language is ambiguous and open to inappropriate moralising and normative pressure that can fail to acknowledge as legitimate the divergent nature of men's (and women's) experiences, values and needs as they prepare to become parents.
